# A Case of Aseptic Meningitis Without Rash Possibly Associated With Varicella Vaccine

**DOI:** 10.7759/cureus.25375

**Published:** 2022-05-26

**Authors:** Yoshihiro Aoki, Toshiyuki Tanaka, Naoto Mizushiro, Katsuhiko Kitazawa

**Affiliations:** 1 Department of Pediatrics, Aizawa Hospital, Matsumoto, JPN; 2 Department of Pediatrics, Asahi General Hospital, Asahi, JPN

**Keywords:** adolescent medicine, serology, aseptic meningitis, varicella vaccine, varicella-zoster virus

## Abstract

Varicella-zoster virus (VZV) may cause aseptic meningitis in the pediatric age group. We describe a pediatric case of aseptic meningitis with a substantial increase of the paired serum antibody to VZV in which the child did not have skin rash during the course of illness. The patient was a 13-year-old boy without any history of exposure to VZV who was admitted with headache, vomiting, and low-grade fever. He had received one dose of varicella vaccine derived from the Oka/Biken strain (vOka) at the age of one year. Cerebrospinal fluid (CSF) analysis on admission revealed an elevated white blood cell count at 609/mm^3^ with 99.6% mononuclear cells. As his symptoms resolved after lumbar puncture alone, he was discharged on the seventh day of hospitalization without receiving any specific medication. Serum VZV-IgG titer was found to be substantially elevated after two weeks. VZV infection and reactivations associated with vaccination, as well as past infections, should be included in the differential diagnoses of pediatric aseptic meningitis, even in the absence of skin rash in the entire course. Polymerase chain reaction (PCR) testing for VZV DNA in CSF should be performed in all cases, if available.

## Introduction

The diagnosis of pediatric aseptic meningitis can be challenging. Most cases are self-limiting and do not require any specific treatment. However, some patients may develop prolonged fever, headache, and vomiting with substantial pain and suffering. Varicella-zoster virus (VZV) may represent an etiology of aseptic meningitis or encephalitis in children, usually presenting with a vesicular rash that follows a dermatome [1]. In clinical settings, the test for VZV with cerebrospinal fluid (CSF) samples of aseptic meningitis patients is sometimes not available or omitted, especially for those without rash. In this report, we present a child with aseptic meningitis without rash in which serological findings indicated VZV reactivation.

## Case presentation

A 13-year-old boy with a history of migraine and asthma presented to the emergency department (ED) with a headache, vomiting, and low-grade fever that had started three days prior. He had received one dose of varicella vaccine derived from the Oka/Biken strain (vOka) at one year of age. He had no history of VZV infection, and no epidemic or contact history of VZV infection. He had visited the ED one day before the current admission, complaining of headache accompanied by nausea, vomiting, and low-grade fever. However, he had been discharged with analgesics as his physical examination and CT findings had been unremarkable. Nevertheless, with no improvement in his symptoms, he revisited the ED the next day. He had no respiratory symptoms, abdominal pain, diarrhea, altered consciousness, abnormal behavior, or paralysis of the face and limbs. Physical examination revealed a temperature of 37.1 °C, heart rate of 65 beats/minute, blood pressure of 113/60 mmHg, respiratory rate of 16 breaths/minute, and oxygen saturation of 98% on room air. His face was pallid; he showed neck stiffness but there was no body rash.

**Figure 1:**
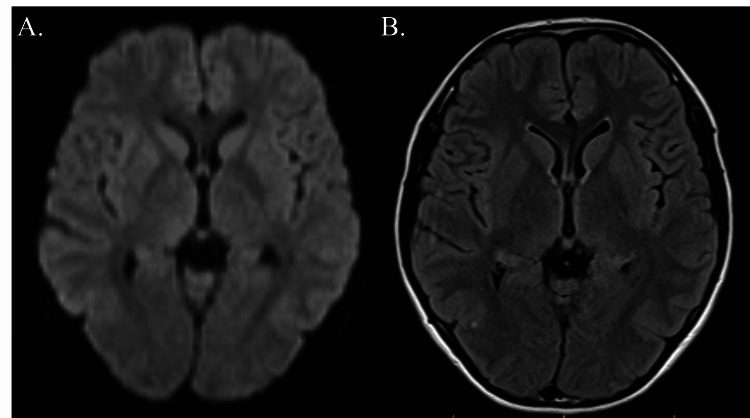
Brain magnetic resonance imaging (MRI) performed on admission (A) Diffusion-weighted MRI and (B) fluid-attenuated inversion recovery (FLAIR) MRI showing no remarkable findings

Blood examination (Table 1) revealed a white blood cell (WBC) count of 7,340/mm^3^ and a C-reactive protein level of 0.02 mg/dL. Urinalysis revealed an elevated ketone level, and a quantitative antigen test for severe acute respiratory syndrome coronavirus 2 (SARS-CoV-2) returned negative. Brain MRI was unremarkable (Figure 1). CSF analysis showed an opening pressure of 27 cmH_2_O (normal range: 6-18 cmH_2_O), glucose of 45 mg/dL (normal range: 40-70 mg/dL) with a blood glucose of 91 mg/dL at the time of lumbar puncture, protein of 136.7 mg/dL (normal range: 10-40 mg/dL), WBC count of 609/mm^3^ (normal range: 0-5/mm^3^) with 99.6% mononuclear cells and 0.4% polymorphonuclear neutrophils, chloride of 123 mEq/L (normal range: 120-128 mEq/L), pneumococcal antigen negative, and Gram-stain negative for organisms, with slightly turbid appearance. Hence, he was diagnosed with aseptic meningitis.

**Table 1:** The results of complete hemogram, biochemistry, serum electrolytes, and blood gas on admission

Test	Reference range	Result
Hemoglobin (g/dL)	13.7–16.8	15.7
White blood cell count (/µL)	3.3–8.6 × 10^3^	7.3 × 10^3^
Differential (%)		
Neutrophils	45.0–72.0	67.8
Eosinophils	0.8–8.4	2.3
Platelet count (/µL)	15.8–34.8 × 10^4^	26.8 × 10^4^
C-reactive protein (mg/dL)	0–0.14	0.02
Albumin (g/L)	4.1–5.1	4.7
Globulin (g/L)	2.5–3.0	2.6
Total bilirubin (mg/dL)	0.4–1.5	0.8
Aspartate aminotransferase (U/L)	13–30	18
Alanine aminotransferase (U/L)	10–42	12
Creatinine kinase (U/L)	59–248	70
Urea nitrogen (mg/dL)	8–20	11.7
Creatinine (mg/dL)	0.40–0.81	0.58
Sodium (mEq/L)	138–145	140
Potassium (mEq/L)	3.6–4.8	3.9
Chloride (mEq/L)	101–108	102
Calcium (mg/dL)	8.8–10.1	9.8
Glucose (mg/dL)	73–200	91
pH	7.35–7.45	7.393
Bicarbonate (mmol/L)	20.0–26.0	19.9
Base excess (mmol/L)	-3.0–3.0	-4.0
Lactate (mmol/L)	0.4–1.8	1.99

After the lumbar puncture, the patient's headache resolved quickly, and his general appearance became normal. He exhibited several fever spikes (maximum temperature of 38.5 °C), but the fever gradually resolved without any specific treatment, which might be attributed to the natural course of viral meningitis. Acetaminophen was given for occasional headaches and fever during his treatment. He was discharged on the seventh day of hospitalization with complete resolution of fever and no recurrent symptoms. Serum VZV-IgG titer measured by enzyme-immunoassay was substantially elevated from 4.3 (reference: <2.0) on admission to 73.3 after two weeks.

## Discussion

We reported a case of aseptic meningitis with a substantial increase in serum VZV-IgG antibody titer. The patient was eventually managed without antivirals; no skin rash developed throughout the treatment course. As polymerase chain reaction (PCR) testing for VZV DNA in CSF was not performed due to the absence of rash and epidemic or contact history of VZV infection; we could prove neither VZV infection nor vOka reactivation in our case. Kawamura et al. [2] have recently reported a similar case of a previously healthy 15-year-old girl with aseptic meningitis who presented with headache, vomiting, and fever without skin rash, caused by the Oka varicella vaccine. The girl also had no history of varicella infection but had received the varicella vaccine at the age of one year. It is important to note that their study reported no significant increase in VZV-IgG antibody titers in the two-week interval but a high copy of VZV DNA was detected in the CSF. In addition, a loop-mediated isothermal amplification assay for VZV identified the virus as the vOka strain, and the patient was treated with acyclovir for 14 days with a positive outcome. According to Kawamura et al., VZV PCR and empiric antiviral therapy should be considered in cases of pediatric aseptic meningitis, even in patients without rash.

Notably, our patient’s condition resolved after lumbar puncture alone, indicating that antivirals may not always be required for VZV meningitis, which may have a self-limiting course. In our case, we did not suspect VZV meningitis owing to the lack of skin eruption, and nor did we suspect herpes simplex virus encephalitis because of the negative MRI findings; accordingly, no antivirals were administered. The role of antiviral therapy in VZV meningitis management has not yet been established [3], but most cases that were eventually diagnosed as VZV meningitis, regardless of whether skin rash was present, were treated with antivirals due to limited evidence from controlled trials, which are difficult to be conducted due to the low incidence of VZV-related disorders [4,5].

Although the biggest limitation in the current case was that VZV was not proven via CSF PCR, and compatibility with the vaccine strain was not confirmed, it was clinically undeniable that it was a case of varicella vaccine meningitis without rash. VZV meningitis without encephalitis may improve with lumbar puncture alone and may resolve spontaneously without any medication, as in our case; therefore, some similar cases may have been overlooked. However, this issue of a possible underdiagnosis requires further validation from future studies. Although there have been substantial cases of varicella vaccine meningitis in the literature [2,6,7], our case is probably the second case of varicella vaccine meningitis without skin rash. However, VZV reactivations causing aseptic meningitis without rash associated with the past varicella infection have also been reported [1,8]; therefore, even in the absence of rash, in cases of aseptic meningitis, performing VZV PCR is crucial; moreover, if VZV test is positive, additional loop-mediated isothermal amplification assay [2] or metagenomic next-generation sequencing [7] for VZV to differentiate the vOka strain from the wild-type VZV strain is essential to confirm the etiology.

## Conclusions

We discussed a pediatric case of aseptic meningitis without rash, possibly associated with the varicella vaccine. Primary VZV infection and reactivations associated with vaccination, as well as the past infection, should be included in the differential diagnoses of pediatric aseptic meningitis without skin rash in the entire course. Paired serology findings can be used as a reference, but VZV PCR with CSF samples may be warranted for determining the etiology.
